# The longitudinal association between external locus of control, social cognition and adolescent psychopathology

**DOI:** 10.1007/s00127-017-1359-z

**Published:** 2017-03-07

**Authors:** Sarah A. Sullivan, Andy Thompson, Daphne Kounali, Glyn Lewis, Stan Zammit

**Affiliations:** 1 Centre for Academic Mental Health, University of Bristol, Oakfield House, Oakfield Grove, Clifton, Bristol, BS8 2BN UK; 2 Division of Mental Health and Wellbeing, Warwick Medical School, University of Warwick, Coventry, CV4 7AL UK; 3 Division of Psychiatry, University College London, Charles Bell House, 67-73 Riding House Street, London, W1W 7EJ UK; 4 Institute of Psychological Medicine and Clinical Neurosciences, University of Cardiff, Cardiff University School of Medicine, Haydn Ellis Building, Maindy Road, Cardiff, CF24 4HQ UK; 5 CLAHRC West, 9th floor, Whitefriars, Lewins Mead, Bristol, BS1 2NT UK

**Keywords:** Psychotic experiences, Depressive symptoms, Social communication, ALSPAC

## Abstract

**Purpose:**

To investigate the longitudinal associations between social cognitive ability an external locus of control (externality) and adolescent psychopathology.

**Methods:**

7058 participants from a prospective population-based cohort provided data on externality, social communication, and emotion perception between 7 and 16 years and psychotic experiences and depressive symptoms at 12 and 18 years. Bivariate probit modelling was used to investigate associations between these risk factors and psychopathological outcomes.

**Results:**

Externality was associated with psychopathology at 12 (psychotic experiences OR 1.23 95% CI 1.14, 1.33; depression OR 1.12 95% CI 1.02, 1.22**)** and 18 years (psychotic experiences OR 1.38 95% CI 1.23, 1.55; depression OR 1.40 95% CI 1.28, 1.52). Poor social communication was associated with depression at both ages (12 years OR 1.22 95% CI 1.11, 1.34; 18 years OR 1.21 95% CI 1.10, 1.33) and marginally associated with psychotic experiences. There was marginal evidence of a larger association between externality and psychotic experiences at 12 years (*p* = 0.06) and between social communication and depression at 12 years (*p* = 0.03).

**Conclusions:**

Externality was more strongly associated with psychotic experiences. At 18 years change in externality, between 8 and 16 years were associated with a larger increase in the risk of depression. Poor social communication was more strongly associated with depression.

**Electronic supplementary material:**

The online version of this article (doi:10.1007/s00127-017-1359-z) contains supplementary material, which is available to authorized users.

## Introduction

It has been proposed that psychosis is an extended phenotype ranging from occasional psychotic experiences (PEs) and no dysfunction to psychotic disorder with serious dysfunction [1]. Studies report that community PEs (i.e., in non-clinical populations) are strongly associated with later psychotic disorder [2, 3] and share risk factors with psychotic disorder [1].

Prevention of psychotic disorders is desirable, but difficult without accurate identification of those at risk before illness onset. Understanding the development of PEs and the identification of risk factors for PEs may be useful, since these often occur before psychotic disorder onset. There has been considerable debate about the true relevance of community PEs which are frequently co-morbid with depressive symptoms (DSs) [4, 5]. The strong overlap between PEs and DSs [6] suggests that PEs may be an expression of underlying emotional distress, rather than an indicator of psychosis vulnerability. In view of this it is not surprising that many risk factors for PEs are also associated with DSs and vice versa [7]. Unpicking the complex associations between risk factors and PEs which are co-morbid with DSs is important, because it may help detect those at specific risk of later psychosis, inform the development of interventions to reduce psychosis incidence, and be informative for understanding the aetiology and mechanism of PEs. Previously, researchers have compared the strength of associations across studies and reported that risk factors associated with schizophrenia seem to be more strongly associated with PEs than DSs; however, these conclusions may be invalid because of differences in study populations. Others have compared estimates of association within the same study [8, 9], but statistical methods that account for the co-morbidity between PEs and DSs are required to address this question robustly. As far as the authors are aware only one previous study has used joint outcome modelling to investigate specific and shared risk factors between PEs and DSs, which reported that only neuro-developmental risk factors were not shared [7].

Social cognitive ability and locus of control (LoC) may be important. There is evidence that deficits in social cognition and an external LoC styles are associated with psychosis [10–15]. Social cognition (the ability to process and use social information) consists of several inter-connected, concurrent processes; the perception, storage, interpretation and implementation of social information. These processes are used to understand and interpret the actions of others and predict their future goals, intentions, and actions, and in turn to adapt one’s own behaviour. The theories of Frith [16, 17] suggest that the signs and symptoms of schizophrenia can be explained by poor social cognitive ability and a consequent inability to infer the mental states of others. There is some cross-sectional evidence from small studies of an association between certain domains of social cognition and PEs or schizotypy (personality characteristics resembling psychotic symptoms), such as theory of mind [18–21], emotion recognition [19, 22–28], and social perception [19, 25]. Longitudinal studies of social cognition have produced conflicting findings. One found an association between theory of mind at age 5 and PEs at age 12 [29] but not between PEs and the recognition of emotion from faces [30].

LoC is the cognitive approach used to attribute causes to events and is related to social cognition, because it relies on self-awareness. Inevitably, individuals draw on their own belief systems to explain the world around them. An external LoC attributes negative events to external causes which are outside the control of the individual, whereas an internal LoC attributes negative events to flaws within oneself. It has been suggested [31] that an external LoC may be associated with PEs, such as paranoia, where an individual may erroneously perceive a threat from an external agent but no evidence that an external LoC is associated with DSs. There is some cross-sectional evidence of an association between an external local of control and PEs or schizotypy [21, 32]. In addition, two prospective studies [15, 33], one using the same data source as this study [15], found that an externalised LoC is associated with PEs at age 12 and psychotic disorder. The study by Thompson et al. [15] differs from our own, because it only investigated the association between LoC at age 8 with PEs at age 12 and did not investigate the role of co-morbid DSs. The finding that only risk factors with a neuro-developmental origin are specific to PEs [7] may have relevance for the investigation of social cognitive ability and LoC as risk factors, because it has been reported that poor social cognitive ability has a neuro-developmental origin, suggesting that it is specific to PEs rather than DSs [34].

Most previous studies investigating social cognitive ability and LoC and PEs, with the exception of one [33], have focussed on childhood ability and early adolescent PEs. It is possible that social cognitive ability and LoC may be a stronger risk factor for later PEs. Although some social cognitive abilities develop early, others may develop (or become more refined) in adolescence. In addition, the demands on social cognitive ability may be more pressing in adolescence when social functioning becomes more important. Our hypothesis, therefore, is that social cognitive ability and LoC are specifically associated with PEs but not with DSs.

## Aims of the study

To investigate;

1. whether childhood social cognitive abilities and LoC are associated with the early and late adolescent PEs and DSs. We hypothesise that social cognitive ability will be specifically associated with PEs and that an externalised LoC will be positively associated with PEs but not associated with DSs.
2. the time-varying effects of social cognitive abilities and LoC on DSs and PEs.

## Methods

### Avon Longitudinal Study of Parents and Children (ALSPAC)

The study sample consists of ALSPAC http://www.alspac.bris.ac.uk participants, an ongoing population-based study investigating a wide range of influences on the health and development of children. All pregnant women resident in the former Avon Health Authority in South West England with an estimated delivery date of between 1st April 1991 and 31st December 1992 were invited to participate. The children of 15,247 pregnancies were recruited. Of this sample of 15,458 foetuses, 14,701 were live births and were alive at 1 year of age [35]. Among these, 7058 children had at least one outcome of interest (DSs or PEs in the early or late adolescence) or at least one risk factor of interest and were included in the analysis. The sample is representative of those born at that time in the former county of Avon during this period [36].

### Ethics

Ethical approval for the study was obtained from the ALSPAC Ethics and Law Committee and the Local Research Ethics Committee.

### Outcome measures

#### PEs at 18 years

The Psychosis-Like Symptom interview (PLIKSi) [37] is a semi-structured instrument that draws on the principles of standardised clinical examination developed for the Schedule for Clinical Assessment in Psychiatry (SCAN) [38]. This measure is further described in Appendix A.

#### PEs at 12 years

The procedure for rating PEs at this timepoint was identical to that at 18 except participants were asked about experiences since their last birthday. Inter-rater reliability across all interviewers was very good (Kappa = 0.72) according to the standard benchmarks [39].

#### DSs at 18 and 12 years

Self-reported DSs were assessed using the Short Moods and Feeling Questionnaire (SMFQ) [40] which asked about symptoms over the previous 2 weeks. The version of the questionnaire used in this analysis consists of only 12 questions. Question 4 “teenager felt restless” was not used because previous work found that this question was poorly understood [41]. Each question had three possible responses: 0 never, 1 sometimes, and 2 always. The score range for each participant was 0 to 24 and higher scores indicated more DSs. The internal reliability of the SMFQ is good (Cronbach’s alpha = 0.85) [40]. The distribution of scores on the SMFQ is highly skewed with the majority reporting low levels of symptoms; therefore, we dichotomised the scale, defining high levels of DSs by scores of ≥11. This cutoff has been shown to have a high sensitivity and specificity [42] and has been previously applied in community samples [43]. Dichotomising DSs allowed joint modelling of both DSs and PEs, as described in the “Statistical analysis”.

### Risk factors

#### Locus of control measures

##### LoC at 8 and 16 years

The shortened version of the Childhood Nowicki-Strickland Internal-External (CNSIE) questionnaire was used to measure external LoC [44]. Questions were asked during an assessment clinic at age 8. At age 16, the questions formed part of a self-completed postal questionnaire, which has good construct validity and test–retest reliability [44]. An external LoC score was calculated as the number of external responses (externality). Higher scores resent a more externalised style.

To investigate the association with outcomes at 18 years scores, externality at 8 and 16 years were used as risk factors. To investigate the association with outcomes at 12 years, externality at 8 years was used as the risk factor.

### Social cognitive measures

#### Social communication and social skills

This was assessed using the Social Communication Disorders Checklist [45] (SCDC) which was completed by parents on behalf of their children at ages 7, 10, 13, and 16 years. The SCDC has excellent internal consistency (0.93) and high test–retest reliability (0.81) [45]. A mean of the scores at 7 and 10 years was investigated in conjunction with the 12-year-old outcomes, and a mean score of the 11- and 13-year-old measures was investigated in confunction with the 18-year-old outcomes.

#### Emotion perception: facial emotions at age 8

Data were collected using the Diagnostic Analysis of Non-Verbal Accuracy (DANVA) [46] during an annual assessment clinic held when the cohort were approximately 8 years. The faces subtest of the DANVA consists of 24 colour photos of male and female primary school age child faces, with each face showing fear, happiness, sadness, or anger. The photos were presented for 2 s after which the participant was asked to respond by indicating which emotion was displayed. Pictures were classified as high (easy to identify) or low (harder to identify) intensity. The test was computerised and exhibits good internal consistency reliability (0.68–0.88) and good test retest reliability (0.70–0.86) [46]. In these analyses, the number of errors made for each emotion with high- and low-intensity stimuli was used.

#### Emotion perception: biological movement at 12 years

This was assessed during the same clinic as the PLIKSi using the Emotional Triangles test [47]. This computerised test consists of silent animations with a black outline triangle and circle on a white background. The movements of the animated shapes were designed to represent the way a person might move if affected by the target emotion. For instance, scared was depicted by the triangle ‘jabbing’ at the circle. The shapes appeared to interact in a manner appropriate to one of four basic emotions; happy, sad, angry, and scared. For each of the four emotion trials, two different types of questions are asked: one that is emotion-appropriate (“is the triangle happy?” for a happy animation) and one that is not (“is the triangle happy?” for a sad/angry/scared animation). Inappropriate responses were subtracted from appropriate responses to account for response bias. High scores represent a better performance. This risk factor was only investigated in relation to the 18-year-old outcomes.

### Potential confounding variables

These were chosen using knowledge of variables which may be associated with PEs and DSs in general population samples and from previous relevant research. Those selected were gender, maternal marital, and educational status at the child’s birth, child’s IQ at age 8 collected using the alternate items from the Wechsler Intelligence Scale for Children (WISC) [48] and the number of autistic traits between ages 6 months and 9 years [49].

**Fig. 1:**
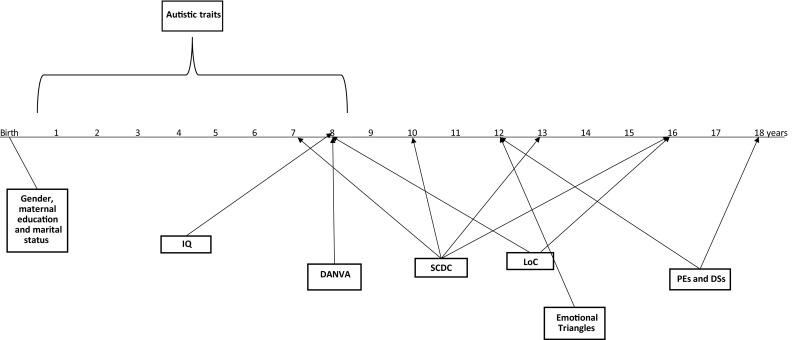
Timeline. A time line of data collection points. Key: *DANVA* Diagnostic Analysis of Non-Verbal Accuracy, *SCDC* Social and Communication Disorders Checklist, *PE* psychotic experiences, *DS* depressive symptoms, *LoC* locus of control

Figure 1 depicts the time line for data collection points.

### Data preparation

#### Missing data

Multiple imputation of missing data was conducted by fully conditional specification using flexible additive imputation models as implemented in the ‘aregImpute’ function in the R statistical package [50, 51] (see Appendix B for further information).

#### Statistical analysis

Standardised versions of risk factor and confounder variables were used for analyses. Initially, the associations between externality and social cognitive ability and PEs at 12 and 18 years and DSs at 12 and 18 years were investigated using logistic regression modelling in imputed data. Multivariable models were used to adjust for confounders and the corresponding psychopathology at each timepoint.

Second, four-dimensional probit regression models were used to jointly model the binary indicators of PEs and DSs at each time-point and to compare regression parameter outcomes using Wald tests after imputation using Stata [52], R [53] and MLwiN [54] software packages. We converted probit estimates into odds ratios to enable interpretability by obtaining approximations of the logit parameters by multiplying the probit parameters by a factor of 1.6 [55].

Joint modelling of depression and PEs at 12 and 18 years allows us to examine whether increases in the levels of social communication, externality and emotion perception could change the risk of depression and PE at each timepoint. The joint modelling approach estimates all these effect simultaneously in a single model and thus allows us to compare these effects (as quantified through odds ratios). The comparison of such effects automatically translates to:

- compare effects between outcomes, e.g., whether externality at 8 years has a similar association with the risk of depression and PE at 12 years and similarly whether externality at 16 years has a similar association with depression and PE at 18 years (in other words whether each exposure is common or specific to each psychopathology) and
- compare effects across time, e.g., whether externality at 8 and 16 years has a similar effect for depression at 12 and depression at 18 years, respectively (in other words, test whether externality has a time-dependent effect on depression or PE).

## Results

### Descriptive data

Participants were slightly more likely to be female and have a mother who was married at their birth who did not have a higher education qualification compared to the overall sample (Table 1). This was as expected from the ALSPAC cohort follow-up [35, 56]. The proportion of those with PEs decreased between 12 and 18 years, whereas the proportion with DSs increased. Imputed data used in this analysis differed little from observed data (Supplementary Table A).

**Table 1:** Description of risk factors, outcome variables, and participants

Measure	Scale	Analysis units	Sample *N*	*N* (%) exposed
Confounders				
Sex	Binary	Female	7058	3645 (51.6%)
Marital status of mother	Binary	Not married	6703	963 (14.4%)
Low maternal education	Binary	CSEs/O-levels	6604	3785 (57.3%)
IQ 8 years	WISC (45–151)	NED (1sd:=15)^(^**^)^	5665	
Autistic traits 6 months–9 years	0–93	NED (1sd:=17)^(^**^)^	6968	
Main risk factors				
Social communication 7 years	0–24	NED (1sd:=3)^(^**^)^	5674	
Social communication 10 years	0–12	NED (1sd:=0.5)^(^**^)^	5631	
Social communication 11 years	0–12	NED (1sd:=0.6)^(^**^)^	5471	
Social communication 13 years	0–12	NED (1sd:=0.7)^(^**^)^	4457	
LoC externality 8 years	0–12	NED (1sd:=2)^(^**^)^	5050	
LoC externality 16 years	0–13	NED (1sd:=2)^(^**^)^	3876	
DANVA 8 years	0–22	NED (1sd:=0.4)^(^**^)^	5390	
Emotional triangles 12 years	(−6) − 20	NED (1sd:=4)	5387	
Outcomes				
DSs at 12 years	(MFQ) 0–25	≥11 score	6343	443 (6.9%)
DSs at 18 years	(CISR) 0–39	≥12 score	4288	655 (15.3%)
PEs at 12 years	(PLIKS) Binary	Presence	6272	859 (13.7%)
PEs at 18 years	(PLIKS) Binary	Presence	4438	411 (9.3%)

^(^**^)^ *NED* Normal Equivalent Deviate—standardised scale. Raw scores are transformed to z-scores (standard normal scores) using the inverse normal function

### Common and specific risk factors

Tables 2 and 3 show unadjusted and adjusted associations between the social cognitive variables and PEs and DSs.

**Table 2:** Results using probit modelling: risk factor effects (OR and 95% CIs) on depression and PEs at 12 and 18 years (*N* = 7058), respectively and averaged over 100 imputations for missing data, as described in “Methods”

	Age	DSs*		PEs*		Wald tests		
						DSs	PEs	Common effect *t* stat. (*p* val.)
		OR	95% CI	OR	95% CI	Time effects *t* stat. (*p* val.)^a^	Time effect *t* stat. (*p* val.)^b^	12 years 18 years^c^
Socio-demographic								
Female	12	1.58	[1.36 1.84]	1.14	[1.00 1.29]	−3.03 (0.002)	−1.64 (0.102)	3.60 (<0.0001)
	18	2.14	[1.85 2.47]	1.32	[1.13 1.54]			4.81 (<0.0001)
Marital status unmarried	12	1.22	[0.98 1.51]	1.32	[1.11 1.56]	−0.62 (0.534)	−2.02 (0.043)	−0.64 (0.525)
	18	1.33	[1.10 1.60]	1.69	[1.37 2.08]			−1.88 (0.060)
Low maternal education	12	1.07	[0.92 1.25]	1.19	[1.04 1.35]	0.07 (0.946)	−1.78 (0.076)	−1.12 (0.263)
	18	1.07	[0.93 1.23]	1.41	[1.20 1.66]			−2.83 (0.005)
Main risk factors								
LoC externality (time-varying)	12	1.15	[1.05 1.25]	1.26	[1.18 1.36]	−3.42 (0.001)	−1.72 (0.087)	−1.93 (0.054)
	18	1.41	[1.29 1.53]	1.41	[1.26 1.58]			
Social communication (time-varying)	12	1.21	[1.10 1.33]	1.08	[1.00 1.17]	0.32 (0.751)	−0.6 (0.546)	1.86 (0.064)
	18	1.19	[1.08 1.30]	1.13	[1.01 1.26]			0.78 (0.433)
DANVA 8 years	12	0.96	[0.89 1.04]	0.92	[0.86 1.00]	0.49 (0.623)	−1.33 (0.184)	0.8 (0.426)
	18	0.94	[0.88 1.01]	0.99	[0.91 1.07]			−1.01 (0.311)
Emotional triangles	12							
	18	1.07	[1.00 1.14]	1.05	[0.97 1.13]			0.34 (0.731)
Confounders								
IQ	12	0.90	[0.83 0.97]	0.86	[0.80 0.92]	−1.81 (0.071)	−0.85 (0.398)	0.92 (0.359)
	18	0.98	[0.91 1.06]	0.89	[0.82 0.97]			1.92 (0.055)
Autistic traits	12	1.08	[1.00 1.17]	1.12	[1.05 1.20]	0.72 (0.469)	−0.55 (0.583)	−0.84 (0.401)
	18	1.04	[0.97 1.12]	1.15	[1.07 1.25]			−2.07 (0.039)

*Analysis scale is expressed in the standard deviation units, where the scale is transformed to have a mean 0 and SD = 1

^a^Test of evidence against the null hypothesis that the strength of the association between risk factors and depression modelled separately at each timepoint is equal to the strength of the association between risk factors and depression modelled across timepoints

^b^Test of evidence against the null hypothesis that the strength of the association between risk factors and psychotic experiences modelled separately at each timepoint is equal to the strength of the association between risk factors and psychotic experiences modelled across timepoints

^c^Test of evidence against the null hypothesis that the strength of the association between risk factors and the psychopathologies modelled separately at each timepoint is equal to the strength of the association between the risk factors and both psychopathologies modelled together at each timepoint

**Table 3:** Main risk factor effects (OR and 95% CIs) on depression and psychotic experiences (PEs) at 12 and 18 years, adjusted for socio-demographic and confounding variables (*N* = 7058), respectively and averaged over 100 imputations for missing data, as described in “Methods”

	Age	DSs*		PEs*		Wald tests		
						DSs	PEs	Common effect *t* stat. (*p* val.)
		OR	95% CI	OR	95% CI	Time effects *t* stat. (*p* val.)^a^	Time effect *t* stat. (*p* val.)^b^	12 years 18 years^c^
Main Risk factors								
LoC externality (time-varying)	12	1.12	[1.02 1.22]	1.23	[1.14 1.33]	−3.57 (<0.0001)	−1.66 (0.098)	−1.87 (0.061)
	18	1.40	[1.28 1.52]	1.38	[1.23 1.55]			0.13 (0.894)
Social communication (time-varying)	12	1.22	[1.11 1.34]	1.06	[0.98 1.15]	0.16 (0.870)	−0.78 (0.438)	2.22 (0.027)
	18	1.21	[1.10 1.33]	1.12	[1.00 1.25]			1.08 (0.282)
DANVA 8 years	12	0.95	[0.87 1.03]	0.90	[0.83 0.98]	−0.21 (0.836)	−1.59 (0.112)	0.93 (0.353)
	18	0.96	[0.89 1.03]	0.98	[0.90 1.06]			−0.45 (0.655)
Emotional triangles	12							
	18	1.09	[1.02 1.17]	1.08	[1.00 1.16]			0.24 (0.808)

*Analysis scale is expressed in the standard deviation units, where the scale is transformed to have a mean 0 and SD = 1;

^a^Test of evidence against the null hypothesis that the strength of the association between risk factors and depression modelled separately at each timepoint is equal to the strength of the association between risk factors and depression modelled across timepoints

^b^Test of evidence against the null hypothesis that the strength of the association between risk factors and psychotic experiences modelled separately at each timepoint is equal to the strength of the association between risk factors and psychotic experiences modelled across timepoints

^c^Test of evidence against the null hypothesis that the strength of the association between risk factors and the psychopathologies modelled separately at each timepoint is equal to the strength of the association between the risk factors and both psychopathologies modelled together at each timepoint

#### External LoC (externality)

In unadjusted analyses, there was evidence that externality at ages 8 and 16 years was associated with PEs and DSs at ages 12 and 18 years. Externality was associated with a higher risk of PEs than DSs and was sustained over time. The association between externality at age 8 years and PEs at 12 years was stronger than with DSs at 12 years, but at 18 years, the risk for DSs had converged to be similar in size. Adjustment for confounding caused some effect attenuation along with larger standard errors, as expected. There was weak evidence of a difference in the strength of the association with PEs across time, where a 1 SD increase in externality at age 8 years was associated with a 23% increase in the odds of PEs at 12 years and a 38% increase at 18 years with externality at 8 and 16 years. There was strong evidence that the association between externality at 8 and 16 years and DSs at age 18 was stronger than that with externality at 8 years and DSs at age 12 with each SD increase in externality associated with a 40% increase in the odds of DSs at 18 compared to a 12% increase at 12 years. There was weak evidence that the association across psychopathologies was different at 12 years (stronger for PEs than DSs) but no evidence of a difference at 18 years, where the risks converged.

#### Social communication

Unadjusted associations showed marginal evidence of an association with PEs at either age, whereas there was evidence of an association between social communication ability at age 7 and 10 years and DSs at 12 years and between social communication ability at 13 and 16 years and DSs at 18 years. After adjustment, there was still only marginal evidence of an association with PEs at either timepoint, however, the association with DSs at both ages remained. At 12 and 18 years, each SD increase in poor social communication ability was associated with an approximate 20% increase in the odds of DSs There was no evidence that the association with either psychopathology differed over time (PEs *p* = 0.44, DSs *p* = 0.87). There was marginal evidence (*p* = 0.03) that the association at age 12 was specific to DSs but no evidence of disorder-specific effects at 18 years (*p* = 0.28).

#### Emotion perception

**Fig. 2:**
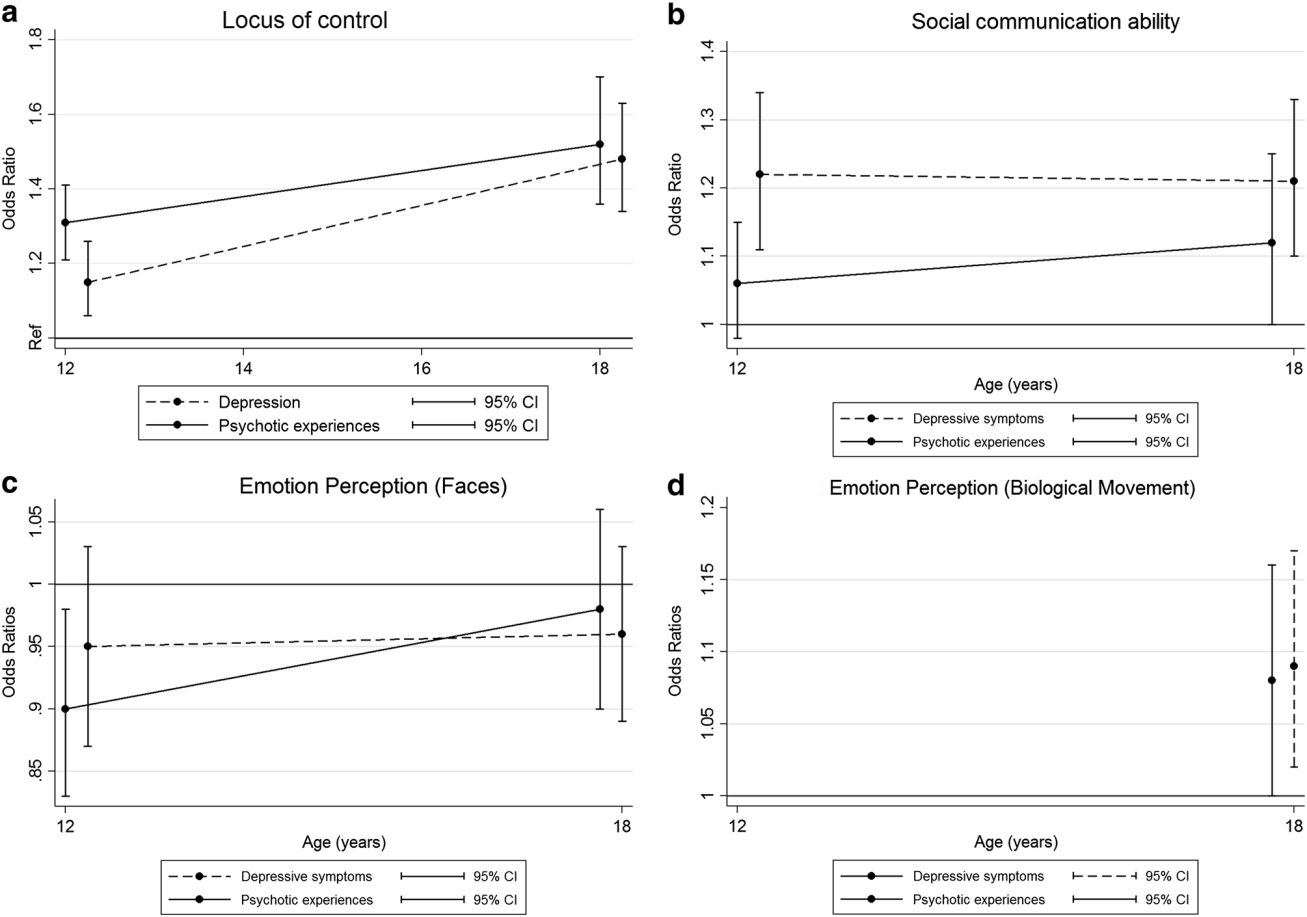
**a** Locus of control, **b** social communication ability, **c** emotion perception (faces), and **d** emotion perception (biological movement)

In unadjusted analyses, there was evidence of a weak association between emotion perception using biological movement at age 12 years and DSs at 18 years but no evidence of an association with PEs at 18 years. There was no evidence of any association between emotion perception ability (facial expression) at 8 years and either psychopathology at 12 or 18 years. After adjustment for confounders, there was weak evidence of an association between emotion perception (biological movement) at 12 years and both PEs and DSs at 18 years. A 1 SD increase in emotion perception ability was associated with a 9% increase in the odds of DSs and an 8% increase in the risk of PEs at 18 years, but the associated uncertainties were large, and could be largely due to measurement error. There remained no evidence of an association between emotion perception (facial expression) and either psychopathology at either age. These findings are illustrated in Fig. 2a–d. The associations between each risk factor and each outcome using logistic regression modelling are described in Supplementary Table B.

## Discussion

Our hypothesis that increased externality and reduced social cognitive ability would be specifically associated with increased risk of PEs was partially supported, since there was some evidence that externality had a stronger impact on the risk of PEs at 12 years than on DSs. However, whilst the association with PEs was time-invariant, it was no longer specific to PEs by age 18. In contrast, there was evidence that social communication deficits were more specific to DSs than PEs at 12 years and there was no evidence that emotion perception was specific to either.

Our findings are in support of the only other longitudinal study on an externality at age 8 and PEs at age 12, which uses data from the same cohort [15]. Our study extends these findings, by simultaneously modelling PEs and DSs in the early and late adolescence. The size of the association detected in this study is larger than our own, which may be because this study did not account for co-morbid depression. Our results suggest that externality is also associated with depression at 12 years, and therefore, a failure to account for this would inflate the estimate. Our findings also support the only other longitudinal study reporting an association between early externality and later psychotic disorder [15, 33]. It is difficult to compare this report of a strong association with ours, since they are expressed as a likelihood ratio Chi-squared for schizophrenia and schizophrenia-spectrum disorder. The difference in the size of associations may be because the outcome was measured in a clinical sample rather than a community sample, or because the participants were from a high-risk group. If psychotic disorders are on a continuum with PEs, associations between risk factors and outcomes are likely to be stronger at the severe end of the continuum. The sample size was also smaller than ours (*n* = 89), and it is increasingly recognised that very small sample sizes can be associated with effect inflation [57]. There was also no adjustment for confounding and, in particular given our findings, of co-morbid depression.

Our findings also expand the previous work in this cohort that investigated the association between emotion perception ability and PEs [30]. This study also failed to find an association.

### Externality, social cognitive ability, and psychopathology

Our findings suggest that externality and poor social communication are potential predictors of later DSs and PEs, even after accounting for co-morbidity and confounding. It is also true that, in general, externality was associated with PEs and poor social communication ability was more strongly associated with DSs. It is easy to speculate how these deficits may lead to each psychopathology. Externality, whereby negative events are attributed to other people or externally-controlled circumstances, may lead to paranoia and delusional belief [31], whereas poor social communication may cause social isolation, leading to DSs. Some psychotic symptoms, such as hallucinations, are associated with a blurring of the boundary of the self, which may be exacerbated by an external LoC which blames self-created negative events on external factors.

It is interesting that in general, associations were stronger for both psychopathologies at 18 than at 12 years, in spite of the fact that PEs are less prevalent in late adolescence. There are several possible explanations for this. It may be that the demands on social cognitive ability are greater in late adolescence, with growing social independence and more complex social tasks. It may also be that PEs at 12 are less accurately measured, perhaps because younger adolescents had difficulty understanding the questions, and therefore, measurement error is diluting the associations between PEs at 12 years and the risk factors. This possibility is endorsed by the lower inter-rater reliability statistics at 12 compared to 18 years. Another possibility is that PEs that have either persisted since age 12, or have been incident since age 12, are of a more severe nature and, therefore, have a stronger association with risk factors.

### The specificity of externality and social cognitive ability as a predictor for PEs

Our hypothesis that externality and poor social cognition would be specifically associated with PEs was based on existing evidence that poor social cognition precedes psychosis, but little evidence that poor social cognition precedes depression [58]. Furthermore, there is a strong evidence that neuro-developmental abnormalities are associated with the risk of schizophrenia [59] and PEs [37].

We found little evidence that associations between externality, social cognitive ability and psychopathology at 18 years were specific to PEs, although at 12 years, there was marginal evidence of this. There are a number of potential explanations. First, PEs in this general population sample may not be strong indicators of aetiological mechanisms underlying psychotic disorders, but may be more representative of emotional distress akin to depression and anxiety; indeed, there is increasing evidence to support this view [60]. Second, it may be that poor social cognition is only a predictor for more severe PEs which are more likely to be associated with later psychotic disorder and there may be an insufficient number of ‘severe’ PEs in our sample. Third, it may only be severe forms of social cognitive dysfunction that are neuro-developmental in origin and there may be few participants with this type of severe social cognitive dysfunction in our sample. Fourth, we used a broad measure of PEs (i.e., both suspected and definite experiences), which may be more similar to DSs than the definite PEs would have been.

It is interesting to speculate why the only indication of specificity was at age 12. It is possible that at the earlier timepoint, there is less overlap between PEs and DSs and that this difference emerged, in spite of the greater difficulty of measuring PEs at 12 compared to 18 years. If it had been possible to measure PEs at the earlier timepoint with even greater accuracy, we may have been able to detect specificity with greater precision.

### Strengths and limitations

To our knowledge, this is the first study to simultaneously model PEs and DSs in the early and late adolescence. This has enabled us to explore the complex relationship between these psychopathologies and the specificity of externality and social cognition as risk factors using a robust statistical method.

This study uses prospective data from a large cohort of adolescents from a general population sample which is representative of the UK population of adolescents [35]. Multiple imputation was used to replace missing data, which has helped to reduce the effect of selection bias resulting from the loss to follow-up of cohort members.

Some limitations of this study should also be acknowledged. There is no measure of PEs before 12 years, which makes it impossible to be certain that the risk factors preceded PEs. However, it should also be acknowledged that measurement of PEs in young children is unlikely to be accurate. The assessment periods for each timepoint of the PEs interviews were different. At 12 years, the participants were asked about any experiences since their 12th birthday (i.e., over the previous 6 months), whereas at 18 years, they were asked about experiences since their 12th birthday (i.e., over 6 years). It is difficult to know what difference this methodological weakness may have had. Potentially, the accuracy of the recall of the participants could have been better at 12 years, as they were being asked to recall experiences over a shorter period. This should have reduced measurement error and thereby increased the probability of detecting an association between risk factors and the 12-year outcomes. However, the probability of recalling an experience may have been greater at the 18-year interview as the participants were asked about experiences which may have occurred over a much longer period, especially if the experiences only happened infrequently. This could have resulted in the recording of a greater number of outcomes, which would also have increased the statistical power to detect an association between risk factors and outcomes. Another limitation is that there were no data recorded in the cohort on confirmed physician diagnosis of either psychosis or depression at 12 or 18 years, although these variables have been subsequently derived from the data by other authors [3]. This is a less important limitation, however, since the aim of this study was to investigate risk factors for PEs and DSs rather than for actual diagnoses, which are in any case likely to be very infrequent at these ages.

Some of the social cognitive measures used here are blunt and may not provide an accurate measure of ability. An additional source of risk factor measurement error may be because some data was collected from parental report (social communication). There are also no data on theory of mind ability in the ALSPAC cohort. It would have been interesting to investigate this ability as a risk factor, since there is convincing evidence that it is associated with psychosis [10, 12].

The importance of PEs as a predictor of psychotic disorder is still undecided, and in fact, most PEs will resolve or not lead to psychotic disorder. However, they are still of importance, since they are a reflection of emotional distress in adolescents and are associated with poor functioning.

### Implications

If our results reflect the causal effect of externality and social communication deficits, then therapies aimed at improving these abilities may reduce the incidence of both PEs and DSs and potentially other psychopathologies, such as anxiety, in early and later adolescence. There is evidence that LoC is modifiable [61] and evidence that interventions to improve social cognition in children may be effective [62].

### Electronic supplementary material

Below is the link to the electronic supplementary material.

Supplementary material 1 (DOCX 14 KB)

Supplementary material 2 (DOCX 12 KB)

## Appendix A: The psychosis-like experiences interview (PLIKSi)

PLIKSi includes 12 ‘core’ questions eliciting key psychotic experiences (PEs) occurring since age 12, covering hallucinations (visual and auditory), delusions (spied on, persecution, thoughts being read, reference, control, and grandiosity), and experiences of thought interference (broadcasting, insertion, and withdrawal). Any unspecified delusions elicited were also rated. Cross-questioning was used to establish the presence of symptoms, and coding of items followed the glossary definitions and rating rules for SCAN. Interviewers were psychology graduates who received training in assessment of the SCAN Psychosis Section and in using the PLIKSi. Interviewers rated symptoms as not present, suspected, or definitely present. Unclear responses after probing were always ‘rated down’, and symptoms only rated as definite when a credible example was provided. If an experience was rated as suspected or definite the participant was asked, whether the experiences reported were always attributable to the effects of sleep (hypnopompic or hypnogogic experiences), fever, or substance use (defined as experiences occurring only within 2 h of intoxication with drugs or alcohol). If this was the case, the experience was not rated as present. Interviewers discussed cases with a psychiatrist if it was unclear how an experience should be rated. At regular intervals, interviewer ratings for a sample of recorded interviews were also rated by a psychiatrist to ensure that interviewers were rating experiences correctly. The average kappa value for inter-rater reliability across the time period of data collection was 0.83 which is considered high using the standard benchmarks.

## Appendix B: Data imputation details

The imputation model included approximately 60 auxiliary variables in addition to those included in the analyses that were either associated with missingness or were predictive of DSs or PEs at 18 years to make the basic assumption underlying multiple imputation realistic. These included a wide range of markers of socio-demographic characteristics during the early childhood and historical assessments of most risk factors. Further information is available from the authors. Parameter estimates were averaged over 100 imputed data sets using Rubin’s rules. Wald-type tests were used following imputation to assess equality/commonality of risk factor effects.

**Table:** Pattern of missing data

Variable	Age of assessment	*n*	% Missing
Psychotic experiences	18	4199	51.5
	12	6115	29.4
DANVA	8	6091	29.6
Locus of control		1938	76.8
	16	4337	49.9
Emotional triangles	12	5113	40.9
SCDC	7	2174	74.9
	10	2441	71.8
	13	2442	71.8
	16	1870	78.4
Maternal marital status	Child’s birth	8656	0
Maternal education		8656	0
IQ	8	6009	30.6
Emotion domain of SDQ	7	6488	25.1

*DANVA* Diagnostic Analysis of Non-verbal Accuracy. *SCDC* Social Communication Disorders Checklist. *SDQ* Strengths and Difficulties Questionnaire

### Extra variables used in missing data imputation

Locus of control assessed with the Cognitive Styles Questionnaire at age 18 years.

Psychotic experiences assessed by self-report questionnaire at 11, 13, 14, and 16 years.

Seven autistic traits are verbal ability, language acquisition, semantic-pragmatic skills, social understanding, repetitive behaviour, articulation, and social inhibition, which were derived from a factor analysis of 93 variables consisting of data collected between 5 and 10 years [63].
