# Supplementary material for: The longitudinal association between external locus of control, social cognition and adolescent psychopathology
Source: Soc Psychiatry Psychiatr Epidemiol. 2017 Mar 7;52(6):643–55. doi: 10.1007/s00127-017-1359-z (PMC5487605; doi:10.1007/s00127-017-1359-z)
Supplement: Supplementary file 1 — Supplementary material 1 (DOCX 14 KB) [file 127_2017_1359_MOESM1_ESM.docx]

**Supplementary Table A: Comparing modality statistics between observed and imputed variables (proportions for binary variables and means for continuous variables. These statistics are averaged across the 100 complete datasets using the Rubin’s rule)**

| **Analyses Variables** | **Observed** | |  |  | **Imputed** | **Mean** | **2.50%** | **97.50%** |
| --- | --- | --- | --- | --- | --- | --- | --- | --- |
| **Confounders** |  |  |  |  |  |  |  |  |
| Sex | Binary | Female | 7058 | 3645 (51.6%) | N=7058 | NA | NA | NA |
| Marital status of mother | Binary | Not married | 6703 | 963 (14.4%) | | 14.546% | 13.699% | 15.394% |
| Low maternal education | Binary | CSEs/O-levels | 6604 | 3785 (57.3%) | | 57.929% | 56.741% | 59.116% |
| IQ 8 years | WISC (45-151) | NED (1sd:=16.2) (**) | 5665 |  |  | 0.000 | -0.023 | 0.023 |
| Autistic traits 6 months – 9 years | 0-93 | NED (1sd:=17.2) (**) | 6968 |  |  | -0.001 | -0.024 | 0.022 |
|  |  |  |  |  |  |  |  |  |
| **Main Exposures** |  |  |  |  |  |  |  |  |
| Social Communication 7 years | 0-24 | NED (1sd:=3) (**) | 5674 |  |  | 0.013 | -0.003 | 0.049 |
| Social Communication 10 years | 0-12 | NED (1sd:=0.5) (**) | 5631 |  |  | 0.013 | -0.019 | 0.033 |
| Social Communication 11 years | 0-12 | NED (1sd:=0.6) (**) | 5471 |  |  | 0.014 | -0.017 | 0.036 |
| Social Communication 13 years | 0-12 | NED (1sd:=0.7) (**) | 4457 |  |  | 0.014 | -0.016 | 0.041 |
|  |  |  |  |  |  |  |  |  |
| Locus of control 8 years | 0-12 | NED (1sd:=2) (**) | 5050 |  |  | 0.006 | -0.021 | 0.032 |
| Locus of control 16 years | 0-13 | NED (1sd:=2) (**) | 3876 |  |  | 0.006 | -0.022 | 0.035 |
| DANVA 8 years | 0-22 | NED (1sd:=0.4) (**) | 5390 |  |  | -0.007 | -0.031 | 0.018 |
| Emotional Triangles 12 years | (-6) - 20 | NED (1sd:=4) | 5387 |  |  | 0.000 | -0.023 | 0.023 |
|  |  |  |  |  |  |  |  |  |
|  |  |  |  |  |  |  |  |  |
| **Outcomes** |  |  |  |  |  |  |  |  |
| Depressive symptoms at 12 years | (MFQ) 0-25 | >=11 score | 6343 | 443 (6.9%) | | 7.170% | 6.538% | 7.802% |
| Depressive symptoms at 18 years | (CISR) 0-39 | >= 12 score | 4288 | 655 (15.3%) | | 14.871% | 13.909% | 15.834% |
| PE at 12 years | (PLIKS) Binary | Presence | 6272 | 859 (13.7%) | | 14.151% | 13.294% | 15.008% |
| PE at 18 years | (PLIKS) Binary | Presence | 4438 | 411 (9.3%) | | 9.160% | 8.390% | 9.929% |
| (**): NED: Normal Equivalent Deviate – standardised scale. Raw scores are transformed to z-scores (standard normal scores) using the inverse normal function. These variables are standardized with zero mean and sd=1. | | | | | | | | |
