# Supplementary material for: The longitudinal association between external locus of control, social cognition and adolescent psychopathology
Source: Soc Psychiatry Psychiatr Epidemiol. 2017 Mar 7;52(6):643–55. doi: 10.1007/s00127-017-1359-z (PMC5487605; doi:10.1007/s00127-017-1359-z)
Supplement: Supplementary file 2 — Supplementary material 2 (DOCX 12 KB) [file 127_2017_1359_MOESM2_ESM.docx]

**Supplementary Table B: Results using regression modelling: Exposure effects (OR and 95% CIs) on depression and psychotic experiences (PEs) at 12 and 18 years (N=7058), respectively and averaged over 100 imputations for missing exposure data as described in methods.**

| **Outcomes** |  | **Depression unadjusted** |  | **Depression adjusted^a^** |  | **PEs unadjusted** |  | **PEs adjusted^a^** |  |
| --- | --- | --- | --- | --- | --- | --- | --- | --- | --- |
|  | **Age** | **OR** | **95% CI p** | **OR** | **95% CI p** | **OR** | **95% CI p** | **OR** | **95% CI p** |
| **Social communication** | **12** | 1.34 | 1.18, 1.51 p≤0.001 | 1.33 | 1.17, 1.51 p≤0.001 | 1.14 | 1.03, 1.25 p=0.01 | 1.07 | (0.97, 1.18) p=0.20 |
|  | **18** | 1.26 | 1.13, 1.40 p≤0.001 | 1.25 | 1.12, 1.40 p≤0.001 | 1.20 | 1.05, 1.38 p=0.008 | 1.12 | 0.97, 1.29 p=0.12 |
| **Locus of control** | **12** | 1.21 | 1.09, 1.35 p=0.001 | 1.11 | 0.98, 1.25 p=0.094 | 1.33 | 1.23, 1.45 p≤0.001 | 1.27 | 1.16, 1.39 p≤0.001 |
|  | **18** | 1.64 | 1.45, 1.84 p≤0.001 | 1.54 | 1.35, 1.76 p≤0.001 | 1.86 | 1.60, 2.15 p≤0.001 | 1.61 | 1.36, 1.92 p≤0.001 |
| **Emotion perception: faces** | **12** | 0.95 | 0.86, 1.06 p=0.36 | 0.97 | 0.87, 1.08 p=0.53 | 0.91 | 0.84, 1.00 p=0.05 | 0.89 | 0.81, 0.98 p=0.01 |
|  | **18** | 0.93 | 0.86, 1.01 p=0.08 | 0.95 | 0.87, 1.04 p=0.28 | 0.98 | 0.89, 1.09 p=0.75 | 0.99 | 0.89, 1.09 p=0.79 |
| **Emotion perception: biological movement** | **18** | 0.95 | 1.00, 1.21 p=0.06 | 1.12 | 1.03, 1.21 p-0.009 | 1.10 | 1.00, 1.21 p=0.06 | 1.11 | 1.01, 1.23 p=0.03 |

^a^ adjusted for gender, maternal marital educational status at child’s birth, IQ, number of autistic traits and the corresponding psychopathology at the same time point (i.e. adjusting for psychotic experiences at 12 when depression at 12 is the outcome and adjusting for psychotic experiences at 18 when depression at 18 is the outcome
